# Neutralisation of Local Haemorrhage Induced by the Saw-Scaled Viper *Echis carinatus sochureki* Venom Using Ethanolic Extract of *Hibiscus aethiopicus* L.

**DOI:** 10.1155/2012/540671

**Published:** 2012-05-15

**Authors:** S. S. Hasson, M. S. Al-Balushi, E. A. Said, O. Habbal, M. A. Idris, R. A. A. Mothana, T. A. Sallam, A. A. Al-Jabri

**Affiliations:** ^1^Department of Microbiology and Immunology, College of Medicine and Health Sciences, Sultan Qaboos University, P.O. Box 35, 123, Muscat, Oman; ^2^Department of Human & Clinical Anatomy, College of Medicine and Health Sciences, Sultan Qaboos University, P.O. Box 35, Muscat 123, Oman; ^3^Pharmacognosy Department, College of Pharmacy, King Saud University, P.O. Box 2457, Riyadh 11451, Saudi Arabia; ^4^Department of Community Health, Faculty of Medical Sciences, Al-Baha University, Saudi Arabia

## Abstract

The objective of the study is to investigate the anti-snake venom activities of a local plant, *Hibiscus aethiopicus* L. The *H. aethiopicus* was dried and extracted with ethanol. Different assays were performed according to standard techniques, to evaluate the plant's acute toxicity and its antivenom activities. The results of evaluating the systemic acute toxicity of the *H. aethiopicus* extract using “oral and intra-peritoneal” route were normal even at the highest dose (24 g/kg) tested. All guinea pigs (*n* = 3) when treated with venoms *E. c. sochureki* (75 *μ*g) alone induced acute skin haemorrhage. In contrast, all guinea pigs (*n* = 18) treated with both venom and the plant extract at a concentration between 500 and 1000 mg/kg showed no signs of haemorrhage. Moreover, all guinea pigs (*n* = 18) treated with venom and the plant extract below 400 mg/kg showed acute skin haemorrhage. All guinea pigs treated with venom *E. c. sochureki* (75 *μ*g) alone induced acute skin haemorrhage after both 24 and 32 hours. In contrast, all guinea pigs treated with both venom and the plant extract (administered independently) at concentrations between 500 and 1000 mg/kg showed no signs of haemorrhage after 32 hours. However, after 24 hours all tested guinea pigs showed less inhibition (<60%) compared to that obtained after 32 hours. The outcome of this study reflects that the extract of *H. aethiopicus* plant may contain an endogenous inhibitor of venom induced local haemorrhage.

## 1. Introduction

Envenoming resulting from snake bites remains the most neglected public health issues in many countries, particularly in poor rural communities living in the tropics. *E. c. sochureki* causes numerous deadly bites especially in Asia [1, 2]. Generally envenoming by *Echis* snake vipers is responsible for several clinical complications of severe systemic and local pathology.

Although an intravenous administration of antivenom, prepared from IgG of venom-immunised horses or sheep, is an effective treatment for systemic envenoming [3], the clinical consensus is that antivenom is of limited effectiveness against the effects of local envenoming that develop rapidly after a bite [4]. Such effects include severe pain, oedema, localized haemorrhage, and necrosis [5] which often results in permanent scarring and deformity. The ineffectiveness of antivenom in treating local envenoming has been attributed to the rapid activity of the toxins and the inability of antivenom IgG to cross the blood/tissue barrier [6, 7]. Despite their smaller size, F(ab2)2 and Fab fragments of IgG are also ineffective against the local effects of envenoming, whether administered by intravenous or intramuscular routes [8, 9]. Other limitations of the antivenom(s) also referred to the variability of snake venom. Venom constitution within the same snake species can present considerable geographical variations [10–12], including a diverse in conformity with interpopulational, seasonal, ontogenetic, and individual factors [13]. Therefore, antivenom made against venom of an assured species may not be sufficient against envenomation of the same snake species [14]. Research to develop a treatment for local envenoming against different species as well as among the same species from different areas will be of clinical priority and should focus on the application of natural [15] or synthetic inhibitors [16] against snake venom potent molecules. Plant-derived drugs remain important resource to combat serious diseases. The present study aims to study the antisnake venom activity of a local plant, *Hibiscus aethiopicus *L. which is brought over 200 years ago from Africa and regrown in *Bani-Hushash *region, Sana'a Yemen. This plant was found to be used by traditional healers in Bani-Hushash East of Sana'a to treat some clinical complications including patients bitten by snakes and scorpions. Although *Hibiscus aethiopicus *L. has long been used as a medicinal plant by traditional healers, the validity of the claims made for this plant has been previously tested scientifically by our research group [17].

## 2. Materials and Methods

### 2.1. Plant Material

The whole plant of *Hibiscus aethiopicus *was collected with assistance of a traditional healer, from Bani-Hushaiesh. Authentication and the taxonomic identification of plant materials was confirmed by Dr. A. Wadieh, Department of Botany, Naser College, in Lahj Governorate, University of Aden, Republic of Yemen. One kilogram of the fresh plant was dried under mild sunshine. The dried sample was pulverized and stored in plastic bags. A voucher sample was preserved for reference in the herbarium of the Pharmacy School, University of Science and Technology, Sana'a.

### 2.2. Extraction of Plant Material

The air-dried and pulverized plant (leaves and barks) material (200 g) was extracted with 400 mL ethanol (80%, v/v) with continuous shaking overnight (24 hours). The obtained ethanol extract was filtered and evaporated using a rotary evaporator and freeze dryer to give the crude dried extract. The dried extract was stored at −20°C until tested.

### 2.3. Source of Venoms

The venom of *E. c. sochureki* (India) used in this study was kindly provided by Mr. P. Rawly - The herpetarium, Liverpool School of Tropical Medicine, Liverpool, UK.

### 2.4. Ethics Statement

This study was carried out in strict according to NIH policies outlined in the Guide for Care and Use of Laboratory Animals. All protocols for animal research were reviewed and approved by the Animal Research Ethics Committee (AREC), University of Science and Technology, School of Medicine and Health Sciences (UST), Sana'a, Republic of Yemen (no. AREC2009-08-07). For experiment that involved human plasma an ethics approval from the Human-Ethics Committee (UST-HEC) of the University of Science and Technology, School of Medicine and Health Sciences (UST), Sana'a, Republic of Yemen was obtained in parallel with a signed consent form from healthy donor (no. HEC/hs/n01-2009/0813ust).

### 2.5. Evaluations of Acute Toxicity of *H. aethiopicus* Extract *In Vivo* Using both “Oral” and “Intraperitoneal” Routes

Two *in vivo *assays using both “oral” and “intra-peritoneal” routes were performed to evaluate the acute toxicity and the cumulative effect “safety protection” potentialities of the *H. aethiopicus* composition.

### 2.6. Oral Route Acute Toxicity

Ten guinea pigs (300–900 g) were used in the toxicity profile. The guinea pigs were given different dosages to investigate the lethal dose. The extract solution (dissolved in water) was given orally using anaesthesia at variable dosages to reach 24 g/kg above optimum of 16 g/kg (a lethal dose according to the international standardisation for the classification of substances). The animals were observed for behaviour change, continuously for a period of two weeks after such administration. Observation was conducted hourly at day 1, and thereafter at 4–6 times per day. Subsequently, blood samples for biochemical analysis, alanine aminotransferase (ALT), aspartate aminotransferase (AST), complete blood count (CBC), and gamma glutamyl transpeptidase (GGT) were collected. At the end of the observation period, animals were sacrificed and dissected. Histopathology examination of their eyes, liver, lung, and spleen was performed.

### 2.7. Intraperitoneal Route Acute Toxicity

Eighteen guinea pigs (300–900 g) of both sexes were distributed randomly into 6 groups (each group of 3 animals) and being treated i.p. with increasing dosages (250, 500, 750, 1000, 1300, and 1600 mg/kg) of *H. aethiopicus *extract. The fifth group served as a control and received an equivalent volume of distilled water. Guinea pigs were observed regularly over a period of 24 hours for signs of acute toxicity and death.

### 2.8. Evaluations of *H. aethiopicus* Extract *In Vivo* for Antisnake Venom Activity

#### 2.8.1. Administration of both Venom and Extract after Preincubation

Thirty nine adult guinea pigs of both sexes (500–600 g) were divided into 2 groups. Group 1 (of 3 guinea pigs) was injected with *E. c. sochureki *venom (75 *μ*g/kg) alone, (dose was previously determined to induce 10 ± 2 mm acute skin haemorrhage). Group 2 was divided into 12 equal subgroups (G2.1–G2.12) of three guinea pigs each. All of the subgroups were injected subcutaneously with a mixture of *E. c. sochureki* venom and *H. aethiopicus *extract (50, 75, 100, 200, 300, 400, 500, 600, 700, 800, 900, and 1000 mg/kg) after both venom and extract were incubated in a test tube for 30 minutes. All animals were observed over 24 hours. At the end of the observation period, animals were sacrificed, and their skins were dissected to examine the haemorrhage neutralisation efficacy of the *H. aethiopicus *extract.

#### 2.8.2. Administration of Extracts 3 Hours Prior to Venom Injection

Twenty eight adult guinea pigs of both sexes (500–600 g) were divided into 2 groups. Group 1 (of 4 guinea pigs) was injected with *E. c. suchorecki *venom (75 *μ*g/kg, subcutaneously) alone. Group 2 was divided into 6 equal subgroups (G2.1–G2.6) of four guinea pigs each. All of the subgroups were injected subcutaneously with the same dose (75 *μ*g/kg, subcutaneously) of *E. c. sochureki* venom 30 minutes after *H. aethiopicus *extract was administered orally by gastric intubation at different concentrations (100, 200, 300, 500, 800, and 1000 mg/kg). Animals (14 at each interval time) were sacrificed, and their skins were dissected to examine the neutralisation efficacy of the extract after 24 and 32 hours, respectively.

### 2.9. Coagulant Activity

The plasma coagulation property was determined according to the method of Theakston and Reid (1983) [18] with minor modification. Briefly normal healthy human-citrated plasma 200 *μ*L (preincubated at 37°C) was incubated with 75 *μ*g of venom sample (dissolved in 50 *μ*L PBS, pH 7.2), and the clotting time was recorded against a light source. For inhibition examination, the venom sample was preincubated with the extract at different concentrations (i.e., 100, 200, 400, 500, and 1000 mg).

### 2.10. Oedema-Inducing Activity

The oedema-inducing activity was assayed according to the method of Vishwanath et al., (1987) [19] with minor modification. Group 1 of 3 guinea pigs was injected in the right footpads with 3x minimum oedema dose (MED) (7.5 *μ*g, previously determined) of venom sample (dissolved in 20 *μ*L PBS, pH 7.2). The left footpads received saline, as controls. Legs were cut off at the ankle joint after 6 hours. An increase in weight due to oedema was calculated as the oedema ratio, which equals the weight of the oedematous leg × 100/weight of control leg. The MED was defined as the amount of venom sample required to cause an oedema of 100%. Group 2 (for inhibition examination) was divided into 8 equal subgroups (3 guinea pig in each) were injected subcutaneously with the venom sample that was preincubated with the extract at different concentrations (37.5–525 *μ*g) for 30 minutes at 37°C.

## 3. Results

### 3.1. Administration of *H. aethiopicus* via “Oral and Intra Peritonial” Routes Has no Acute Toxic Effect

All animals were alive after the 2 weeks of given 24 g/kg extract solution above the lethal dosage of 16 g/Kg. No abnormal behaviour was observed. Animals showed normal body weight increase during the two weeks period. Biochemical analysis showed normal range of ALT, AST, CBC, and GGT (Table 1). Inspection of the eyes, liver, lung, and spleen showed no extraordinary signs. The results when compared to a general acute toxicity index were normal, and no acute toxicity was observed. Furthermore, guinea pigs dosed intra-peritoneally with *H. aethiopicus *extract were initially dull with significantly reduced movement for 20–30 minutes. However, neither death nor signs of toxicity were observed even at the highest dose (24 g/kg) tested.

### 3.2. Preincubation of *H. aethiopicus* Inhibits Venom-Induced Haemorrhage

**Figure 1:**
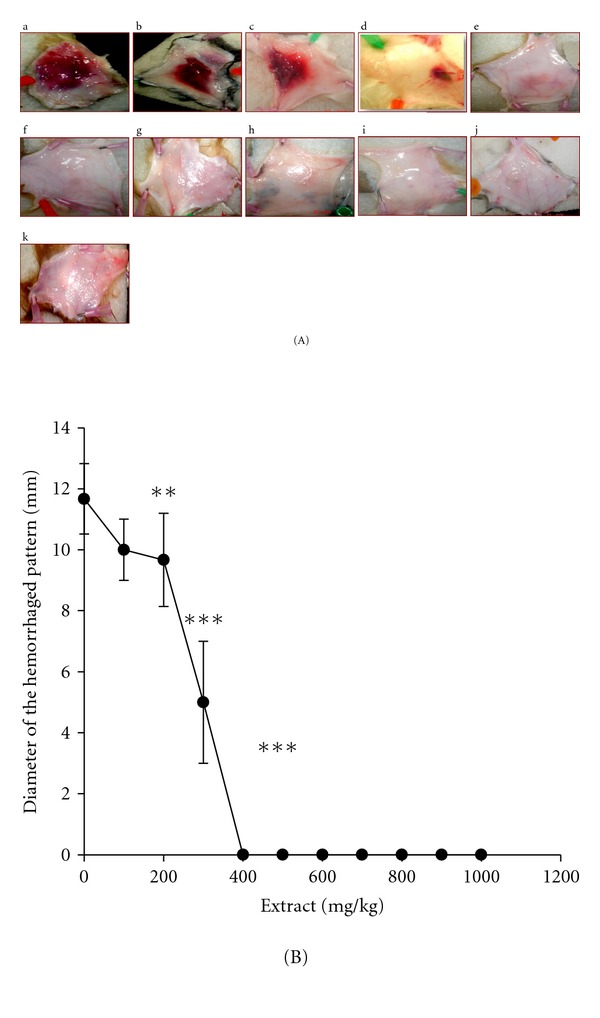
(A) Dissected skins of guinea pigs showing dose dependent of antivenom neutralisation efficacy of *H. aethiopicus extract. *(a) Control: animals were injected with 75 *μ*g of *E. c. sochureki *alone. (b–k) represent different concentration (100–1000 mg/kg) of the *H. aethiopicus *extract. (f) represents the optimum concentration (500 mg/kg) of the *H. aethiopicus *extract that gives a complete venom neutralisation after both venom and extract were incubated in a test tube for 30 minutes. (B) Response curve showing dose dependent of antivenom neutralisation efficacy of *H. aethiopicus extract.* Neutralisation efficacy of different concentrations (100–1000 mg/kg) of the *H. aethiopicus* extract preincubated in a test tube for 30 minutes with fixed concentration of the *E. c. sochureki *venom. Chart illustrates the end point concentration (i.e., 400) where no adequate haemorrhage pattern found to be measured. **P < 0.01 and ***P < 0.0001.

All guinea pigs treated (injected subcutaneously) with venoms *E. c. sochureki* (75 *μ*g) alone “Group 1” (*n* = 3) induced 10 ± 2 mm acute skin haemorrhage (Figure 1(A), a). In contrast, all guinea pigs “Group 2” (*n* = 18) treated with both venom and the plant extract at concentrations between 500 mg/kg (Figure 1(A), f) and 1000 mg/kg (Figure 1(A), k) showed no signs of acute skin haemorrhage (*P* < 0.002) (Figure 1(B)). Moreover, all guinea pigs (*n* = 18) treated with venom and the plant extract below 500 mg/kg showed acute haemorrhage similar to the controls (Figure 1(A), b–e).

### 3.3. Oral Administration of *H. aethiopicus* Prior to Venom Injection Inhibits Haemorrhage Induction

**Figure 2:**
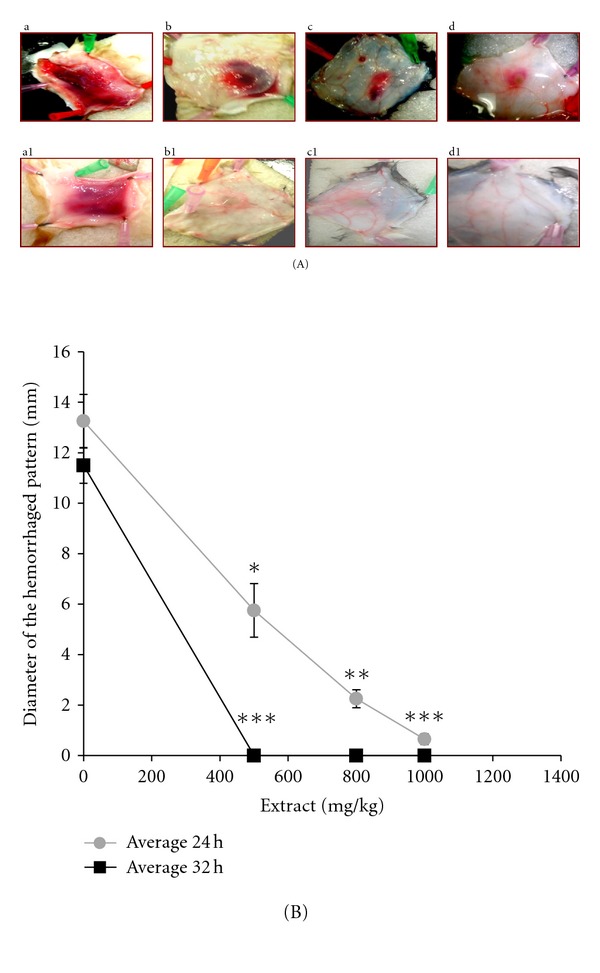
(A) Dissected skins of guinea pigs showing oral administrations of the *H. aethiopicus* extract three hours prior to venom injection (a) control where animals were injected with 75 *μ*g of *E. c. sochureki* alone; (b, c, and d) represent different concentrations (500, 800, and 1000 mg/kg), respectively, of the *H. aethiopicus* extract that was orally administered 3 hours prior to venom injection, and animals were sacrificed after 24 hours to examine their skins; (a1 to d1) are the same of the above except that animals where sacrificed and skins where dissected after 32 hours. (B) Chart showing oral administrations of the *H. aethiopicus* extract three hours prior to venom injection. Neutralisation efficacy of different concentrations of the *H. aethiopicus* extract when administered orally three hours prior to venom injection (75 *μ*g). The grey and black lines illustrate the gradual neutralisation efficacy of the extract correlated to different concentrations after 24 and 32 hours results, respectively, of animal dissected. *P < 0.05, **P < 0.01 and ***P < 0.0001.

All guinea pigs treated (*n* = 4) with venoms *E. c. sochureki* (75 *μ*g) alone “Group 1” induced acute skin haemorrhage (10 mm) after both 24 (Figure 2(A), a) and 32 hours (Figure 2(A), a1) of skin dissection. In contrast, all guinea pigs “Group 2 (*n* = 6)” treated with both venom and the plant extract at concentrations of 500, 800, and 1000 mg/kg showed no signs of acute skin haemorrhage after 32 hours (*P* ≤ 0.0001) (Figure 2(A), b1–d1 and Figure 2(B)) a *P* < 0.0001. However, after 24 hours (Figure 2(A), b–d), all 6 animals showed similar haemorrhage pattern with the control (Figure 2(B), a1) and (Figure 2(B)) a *P* value of 0.001. All guinea pigs treated with venom and the plant extract below 500 mg/kg showed acute haemorrhage (*n* = 12), regardless of the time the skin dissection was taken (data not shown).

### 3.4. The *H. aethiopicus* Extract Inhibits Venom Coagulation Activity

**Figure 3:**
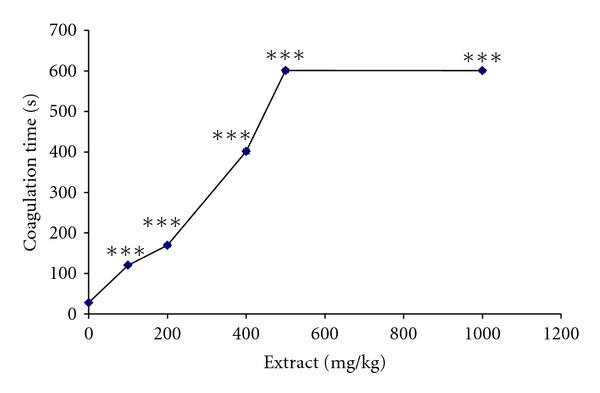
Inhibition of procoagulant activity. Fixed dose of *E. c. sochureki* venom (75 *μ*g) was preincubated with variable concentration of the extract at 37°C for 30 min. Then, 100 *μ*L of the mixture was added to 200 *μ*L of citrated human plasma and the coagulation time was determined. The inset shows the dose-dependent procoagulant activity of *E. c. sochureki* venom on citrated healthy human plasma. ***P < 0.0001.

The extract was found to inhibit, dose dependently, the procoagulant activity of the *E. c. sochureki* venom (Figure 3). A progressive increased clotting time resulting in anticoagulation was recorded (Figure 3). The anticoagulant response reached an optimum with a clotting time of 600 ± 4, and this was attained at the extract concentration of 500 mg (*P* < 0.0001). No significant difference was observed between both concentration of 500 and 1000 mg as both gave the same anticoagulant response. In contrast, in the absence of the extract, the control value containing the venom sample alone revealed a clotting time of 28 ± 3 s (*P *≤ 0.0001). However, when induced with Ca^+2^ alone, clot formation was seen at  325 ± 4 s (data not shown).

### 3.5. The *H. aethiopicus* Extract Inhibits Oedema Induction

**Figure 4:**
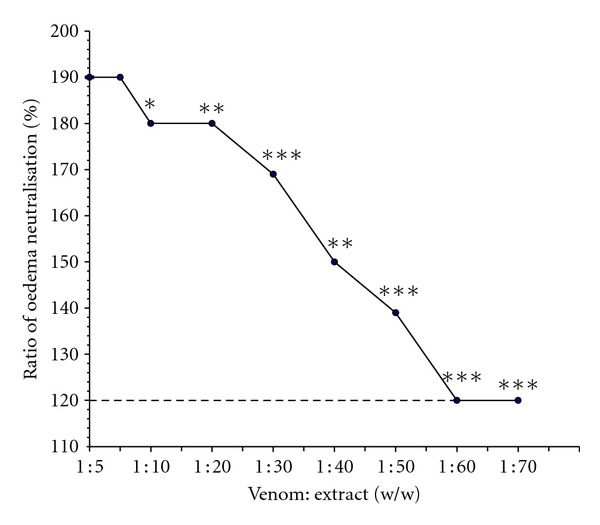
Response curve of dose dependent of oedema neutralization. Experiments were performed by preincubating 37.5–525 *μ*g of extract and fixed doses (7.5 *μ*g) of *E. c. sochureki* venom for 30 min at 37°C (see Section 2). Dotted line represents the control, that is, the foot pad that received a saline only. Data are represented as mean ± SEM (*n* = 3).

Toxicity studies in a mouse model revealed the following. The MED was found to be 7.5 *μ*g (this is the amount of venom sample required to cause an oedema of 100%). The 3x MED found to cause an oedema of 190 ± 2%. The venom-induced haemorrhagic oedema was also inhibited dose dependently by the extract. At a ratio of venom to extract of 1 : 60 and 1 : 70 (w/w), the oedema of 190 ± 2.0% (caused due to 3x MED of venom) was reduced to 120 ± 2.0% which is the same as the control (i.e., leg injected with PBS), (*P* < 0.0001) (i.e., the foot pad that was injected with the saline alone) (Figure 4).

## 4. Discussion

Plants constitute rich sources of novel compounds with a variety of pharmacological activities. Therefore, experimental validation of the traditional use of plants is important and can facilitate the development of low-cost phytotherapeutic agents [9]. Plants used as remedy for snakebite abound in literature [20–23]. However, many of the reported studies lack detailed scientific investigation, which is needed in the development of medicinal agents from plants [20–22].

In this study we have used *H. aethiopicus, *one of the main traditional herbal plants which is used as phytotherapy practiced by a large proportion of the Yemen population for the treatment of several clinical complications including snake envenomation. To our knowledge no scientific reports on *H. aethiopicus *and its capacity to neutralise snake venom(s) available except a single study that was published by our research group [17]. Therefore, this study represents the second report about *H. aethiopicus *and its uses as antivenom agent against *E. c. sochureki*.

In contrast to our results reported in the previous work [17], the oral route when compared to a general acute toxicity index showed normal with no extraordinary symptoms as well as no acute toxicity. However, both routes showed no death even at the highest tested dose (24 mg/kg) compared to the previous work. This was supported further by the biochemical analysis as shown in Table 1. However, with intra-peritoneal route doses, guinea pigs were initially dull with significantly reduced movement for about 20–30 minutes (*P* < 0.005).

In the subsequent experiments we used the *H. aethiopicus *extract to assess its efficacy to neutralise the haemorrhagic activity of *E. c. sochureki* venom using an *in vivo* minimum haemorrhagic dose (MHD). It was interesting to note that results of the evaluation assays of antisnake venom activity showed that *H. aethiopicus *induced noticeable and significant neutralisation capacity against venom of *E. c. sochureki. *This was clearly illustrated in Figure 1(A), a–k. In a guinea pig animal model, MHD was found to be 25 *μ*g, and the 3x MED was found to cause the skin haemorrhagic area of 10 ± 2 mm. It can be seen from (Figure 1(A), e) that at concentration of 400 mg/kg which act as the end point (Figure 1(B)) showed a slight haemorrhage and/or inflammation. Therefore, above, that is, 400 mg/kg, neutralisation efficacy of 100% was achieved at a concentration of 500 mg/kg (Figure 1(A), f).

Results from the oral administration of the extract 3 hours prior to venom injection were significant and showed future promising (Figure 2(A)). In this assay we examined the neutralisation efficacy of the extract using two prospects based on time intervals of 16 and 32 hours, respectively.

In comparison with the results from above we have found that the concentration that gave 100% neutralisation was 1000 mg/kg after 32 hours rather than after 16 hours, suggesting that this difference is due to the time needed for the absorption and distribution of the active components in *H. aethiopicus* extract. Although such comparison between the two assays is not adequate as in the first assay, a close contact between the extract and venom was achieved to give a maximal neutralizing effect. Therefore, further investigation of the absorption rate should be performed. Moreover, this result can also suggest that having the extract before envenomation can act as a prophylactic agent.

Ecarin is an extensively characterized metalloprotease present in *E. c. sochureki* venom and has been found to activate prothrombin directly resulting in the pro-coagulation of citrated human plasma [24]. This property of *E. c. sochureki* procoagulant was found to be independent of Ca^+2^ requirements [25, 26]. In addition, ecarin also cleaved human fibrinogen and dissolved the fibrin clot [27]. The prothrombin converting and fibrinogenolytic (thrombin-like) activity of ecarin appears to be responsible for the procoagulant activity of the venom. Interestingly in this study we found that the extract can inhibit dose dependently the procoagulant activity of the *E. c. sochureki* venom and progressively increases clotting time resulting in anticoagulation (Figure 2(A)). The anticoagulant response of the extract reached an optimum with a clotting time of 600 ± 4 s, and this was attained at the extract's concentration of 400 mg (*P* < 0.0001). However, no significant difference between both concentrations of 500 and 1000 mg was found as both gave the same anticoagulant response. In contrast, in the absence of the extract, the control value containing the venom sample alone revealed a progressively decreasing clotting time which reached an optimum at 28 ± 3 s for a venom concentration of (75 *μ*g) of the venom sample, (*P* < 0.0001) (Figure 3). However, when induced with Ca^+2^ alone, clot formation was seen at 321 ± 4 s (data not shown). Since ecarin appears to be the principal procoagulant agent of *E. c. sochureki* venom, the extract might induce the inactivation of ecarin. This was confirmed by early studies where several anticoagulants have been isolated and studied extensively from this venom [24, 28–31]. In addition to the coagulation assay, we examined the neutralisation of the extract against the oedema inducing activity of the *E. c. sochureki* venom. The result was significant, and the extract was found to inhibit dose dependently the venom-induced haemorrhagic oedema. At a ratio of venom to extract of 1 : 60 and 1 : 70 (w/w), the oedema of 190 ± 2.0% (caused due to 3x MED of venom) was reduced to 120 ± 2.0% which is the same as the control (i.e., the foot pad that was injected with the saline alone) (Figure 4), (*P* ≤ 0.0001). In several experimental and clinical trials, it has been demonstrated that antivenoms are of limited value to stop oedema progression within the first 12–24 hours of treatment, while they are highly efficient in restoring blood coagulation status within the same time interval [32–34]. Moreover, the rapid neutralization of venom in the bloodstream does not guarantee the halting of oedema progression within a short time. Therefore, in this study, we report for first time that the extract of the *H. aethiopicus *showed high neutralizing potency against such effect within six hours of venom injection. However, this finding was based on *in vitro* testing, (i.e., close contact between the extract and venom). Therefore, to confirm such efficacy, an *in vivo* assay has yet to be performed where, the extract has to be given orally “independent,” that is, before and/or after the venom being injected.

Despite these protective effects of the plant extract of the *H. aethiopicus in vitro*, the results obtained from the *in vivo* experiment (Figure 2(A)) were highly encouraging. The extract did protect animals challenged with local haemorrhage of the *E. c. sochureki* venom when the extract and venom were administered independently. However, haemorrhage induction was significantly reduced (*P* < 0.0001) and or fully neutralised with the increase of the extract concentration and time, in contrast with the preincubation assay represented by Figure 1.

The outcome of this study reflects that the extract of *H. aethiopicus *plant may contain an endogenous inhibitor of venom-induced local haemorrhage. This obviously would need further investigations for both systemic and local evaluation. Further studies on the fractionation(s), isolation and characterization of the active principle/s, and its antivenom property appears promising and could contribute to the development of a potent and perhaps safe antidote against *E. c. sochureki* venom poisoning and might play a role in the better management of the threatening lethal bites.

## 5. Summary

Snake bite remains a public health problem in many countries even though; it is difficult to be precise about the actual number of cases. It is estimated that the true incidence of snake envenomation could exceed 5 million per year and causing about 125,000 deaths each year, predominantly within poor communities living in rural areas of countries in Southeast Asia and Africa. Although an intravenous administration of antivenom, prepared from IgG of venomimmunised horses or sheep, is an effective treatment for systemic envenoming, the clinical consensus is that antivenom is of limited effectiveness against the effects of local envenoming that develop rapidly after a bite. In addition to its high cost and other limited effectiveness, thus, there is a need to develop novel therapeutics to maximise the utility of the snakebite therapies that are available. Research to develop a treatment for local envenoming is therefore a clinical priority and has focused on the application of natural or synthetic inhibitors of snake venom potent molecules. The traditional medicine to treat snakebite victims still plays an important role in the primary health care worldwide, especially in rural areas and poor communities in the third world countries is a common practice. Because natural products of higher plants may give a new source of medication, there are many research groups that are now engaged in medicinal plants research not only for the discovery for new drugs, but possibly for discovering compounds with novel mechanisms of action that can stimulate new fields of research. Our results suggest that that *Hibiscus aethiopicus *L. plant may contain an endogenous inhibitor of venom-induced haemorrhage.

## Figures and Tables

**Table 1:** Biochemical analysis for acute toxicity of the *H. aethiopicus* plant.

Group (G)	ALT (Mean^*∗*^)	AST (Mean^*∗*^)	GGT (Mean^*∗*^)
G1: Normal (*n* = 4)	49 ± 3.6^*∗*^	109 ± 2.1	3.2 ± 1
G2: Intoxicated control (*n* = 4)	81 ± 1.8	197 ± 4.1	3.6 ± 0.2
G4: Guinea pigs (*n* = 10)	51 ± 3.2	111 ± 1.3	3.4 ± 0.9

ALT: alanine aminotransferase.

AST: aspartate aminotransferase.

GGT: gamma glutamyl transpeptidase.

Data are expressed as International Units (IU/mL); ^*∗*^Mean value significantly different (*P* < 0.05) compared with respective values (before treatment) using paired Student's *t*-test. *n*:  number of animal (guinea pigs). ^*∗*^All values presented are means *± *SE (standard error). No statistically significant differences were observed.
